# Notice of the Hooping Cough and Measles Appearing Together in the Same Patient, as It Occured at Newport and Its Vicinity, Herkimer Co. N. Y.

**Published:** 1831

**Authors:** A. B. Bowen


ART. V.—*Notice of the Hooping Cough and Measles appearing together in the same patient, as it occurred at Newport and its vicinity, Herkimer Co., N. Y.* By A. B. BOWEN, M. D.

The spring of 1830, in the vicinity of Herkimer County, was uncommonly mild and pleasant, so that, by the last of April, vegetation was nearly two weeks in advance of the seasons. This was checked, however, about the first of May, by one of those sudden changes of weather, so peculiar to the northern and middle states, from warm and pleasant to cold and wet, which latter state continued until the middle of June.

Hooping cough prevailed extensively in Newport, as early as the middle of April, but did not spread much in the country around, till some weeks later; attacking, in its course, almost every individual susceptible of the contagion. There was no peculiarity attending the complaint, or distinguishing it from common pertussis, other than the severity of its symptoms; viz: high fever, distressing cough, almost to suffocation, attended with the whoop, and, in several instances, terminating in epileptic convulsions.

In May, measles made its appearance, attacking a great many of the little patients laboring under cough. In most instances, the active symptoms of the latter complaint had subsided, previous to the measles setting in; and in all with which I was acquainted, the *cough commenced first.*

The attack of measels was usually ushered in by the common premonitory symptoms, and speedily followed by oppression and laborious respiration, arterial excitement, dyspnœa, livid countenance, eruption, and attended with paroxysms of pertussus, aggravated, and distinct from the hoarse cough of measles. We give one case, a fair specimen of many.

*July 4th.* Called 5 o'clock this morning to see a child of Mrs K. A. E. aged 18 months, in convulsions, after a distressing fit of coughing. Attacked about the middle of June; cough dry; stomach irritable; tongue covered with a light brown fur; pulse frequent, not hard or wiry; surface, particularly the extremities, cold; bowels costive.

Warm bath relieved the convulsions; ordered cal. 6 grs., followed in 2 hours by ol. R.

5th. Cal. operated slightly; oil. not given; shehad considerable fever through the night, but less this morning. Ordered the cal. and ol. R. repeated; afternoon, anothe rconvulsion; cath. had not operated; gave a table spoonfull ol. R., which brought away dark fœtid stools in abundance.

6th. Child better; ordered ol. R. next day, and left.

26th. Called again; found her broke out with measles; had coughed as often as once in an hour or two since 6th inst., but not alarmingly; eyes red and suffused; had hot and burning skin; hoarse cough, with dyspnœa and frequent paroxysms of pertussus, distinct and severe; pulse full, hard, and frequent. Bled her 3 oz. in the jugular vein, which relieved her breathing, and gave her an emetic, which threw off much bilious matter, mixed with mucous.

27th. Paroxysms of cough and dyspnœa more distressing, with much hoarse cough and fever; opened her bowels with ol. R., and put her into a warm bath, which relieved her breathing a little; increased again this afternoon, and the system evidently giving way; died at 2 o'clock, A. M.

The commencement and course of this case, was similar to a considerable number of others which came within my observation. Where the system was not too much worn out by the first disease, the termination was in most instances favorable; but, as in this case, when the cough had been of much duration, or when the excitability of the system was worn out from its severity, the complaint was almost surely fatal.

How far these cases go to contradict the observation of the celebrated Mr. Hunter, viz, that two diseases cannot exist together in the same patient, it is perhaps difficult to determine. At first, I could not regard it but as a complete refutation of that doctrine. On reflection, however, I came to the conclusion, that whatever of a *specific* nature there was in the cough, had exerted its influence, and, if the phrase be allowed, spent itself upon the system previous to the attack of measles; that the distinct paroxysms of cough, were the sequelæ of hooping-cough, kept up by habit, and not the immediate effect of the specific agent. In a number of cases where the patients recovered, and all appearance of measles subsided, the cough was kept up for months after, as is common after pertussus.

It may not be improper, although aside from the object of this notice, to mention, in this place, a remedy which was resorted to for the cure of this sequel of pertussis, with happy effect in almost every instance in which it was used; viz: Fol. conii, either in the form of powder or pills, given in grain doses to children, and increasing it until some effect upon the system is produced.

